# Photodynamic therapy and end-stage tongue base cancer: short communication

**DOI:** 10.1186/1758-3284-3-49

**Published:** 2011-12-07

**Authors:** Waseem Jerjes, Tahwinder Upile, Hani Radhi, Colin Hopper

**Affiliations:** 1 Department of Surgery, University College London, London, UK; 2 Oral and Maxillofacial Surgery Unit, AL-Mustansirya University, Baghdad, Iraq; 3 Academic Department of Trauma and Orthopaedic Surgery, School of Medicine, University of Leeds, Leeds, UK; 4 Department of Otolaryngology/Head and Neck Surgery, Chase Farm & Barnet NHS Trust, Enfield, UK; 5 Head & Neck Unit, University College London Hospital, London, UK

## Abstract

We previously reported on the outcome of 21 patients with stage IV advanced and/or recurrent tongue base carcinoma subjected to mTHPC-PDT. We continue to develop on the previous work by treating more patients with this unforgiving disease. PDT has shown to be a very successful minimally-invasive surgical tool in managing this pathology. Tumour-associated symptoms were reduced significantly. The overall morbidity and mortality following PDT, in this group of patients, were far less when compared with other conventional modalities.

## Background

We reported, in a preliminary study, on the use of PDT as a minimally-invasive surgical intervention for advanced and/or recurrent tongue base carcinoma [1]. Twenty-one patients with this unforgiving disease were subjected to mTHPC-US-guided interstitial PDT, the cohort was followed-up for a mean of 36 months (min. 21, max. 45). Tumour response to PDT was remarkable with the majority of the patients reporting reduction of tumour-associated symptoms (P<0.001) (i.e. breathing, swallowing and speech problems). This was confirmed clinically and radiologically. Mortality figures showed that 4 patients died from loco-regional disease spread and further 2 from an existing distant tumour spread.

### Aims

In order to encourage evidence-based practice in surgery, and to ensure that the treatment and processes are sound, we developed upon the work of the preliminary study, collecting data from a larger recruit (33 patients in total). Patients' reports on quality of life with clinical and radiological evaluation were used to assess the outcome.

## Materials and methods

All the 33 (23 males and 10 females) treated patients with advanced and/or recurrent tongue base carcinoma (stage IV) were discussed at the UCLH multidisciplinary meeting. The mean age was 77.5 +/- 8.3 years (min. 48, max. 92) with 90% being Caucasians.

The patients' main complaints were categorized into three parameters: breathing, swallowing and speech problems. Problems associated with pathological growth in the oro-pharyngeal/laryngeal regions caused breathing problems in 14 patients, swallowing problems in 33 patients, and speech (voice) problems in 18 patients. As there is no current verified assessment of quality of life in patients undergoing PDT, the patients were asked only to report the nature of the complaint and not the severity

It was agreed to offer interstitial PDT (iPDT) under general anaesthesia, using mTHPC as the photosensitising agent. mTHPC 0.15 mg/kg was administered into the mid-cubital vein 96 hours prior to treatment. Also, 35 metastatic cervical lymph nodes were identified and treated.

Intra-operatively, an ultrasound probe was used to examine the centre and periphery of the pathological tissue assessing volume, depth, invasion of vascular structures, hollow organs and/or hard tissue. This was followed by insertion of 18 Gauge 70 mm long spinal needles under US-guidance into the target tissue.

Iso-illumination treatment plan was carefully implemented and supervised by a senior physicist. A four-channel 652 nm diode laser was used for illumination. Bare polished-tip laser-light delivery fibres, with a core diameter of 400 μm, were introduced through the spinal needles into the tumour. Light was then delivered from the fibres to the target tissue at 20 J per site (cm^2^).

Postoperatively, gradual light re-exposure and airway and pain control was the priority. Twelve of our patients underwent elective tracheostomy prior to iPDT and 8 others had nasopharyngeal tube inserted. Patients were discharged from hospital care at a mean of 10 days (Min 7, max 22) postoperatively. Patients were followed-up and asked to report on the outcome of their therapy. Clinical assessment outcome was performed by a team of surgeons/physicians trained in photodynamic therapy at approximately 6 weeks postoperatively. MR imaging was performed 5-6 weeks postoperatively. Patients were followed-up for a mean of 48 months (Min 22, Max 72).

### Statistical Analysis

This was performed using the SPSS 17 program (statistical package for social scientists) by an independent statistician. The patients' data were entered onto proformas which were validated and checked by interval sampling. The fields included a range of clinical, operative, and radiological parameters related to the outcome following PDT. The results were cross tabulated and the Chi-squared statistic was used to test for differences in the incidence of outcome. Fisher's exact test was used for the analysis of contingency tables and therefore to measure the P-value. Cross tabulation was also used to compare complications, survival, and cause of death to clinical and radiological assessment.

## Results

The majority of the patients (11/14) reported improvement of breathing (P<0.001), with one patient reporting worsening of symptoms. An improvement of swallowing was reported by 28/33 patients (P<0.001); while speech improvement was evident in 15/18 patients (P<0.001). Clinical assessment showed that two-thirds of the patients had "good response" to the treatment and a third reported "moderate response".

Radiological assessment comparing imaging 6-week post-PDT to the baseline showed stable pathology with no change in size in 6 patients, minimal response (<25% reduction) in 7 patients, moderate response (<50% reduction) in 12 patients and significant response (50-75% reduction) in 8 patients. Thirty-five cervical lymph nodes were subjected to PDT and most of them (P<0.001) become necrotic with either no change or reduction in size.

About 90% of our patients reported no problems following PDT. Unfortunately, due to the extended duration of skin photosensitization following treatment, skin burn was reported by six of our patients; while two patients had skin necrosis caused by treating pathologies very close to the surface.

Fourteen patients needed only one round of iPDT to shrink and control the disease and prevent further progression, while the rest needed two to three rounds of treatment. Follow-up at a mean of 48 months (Min 22, Max 72). revealed eleven mortalities. Cancer-related mortalities included seven from loco-regional disease spread and two from ongoing distant metastasis.

## Conclusion

In the current short communication, the cohort was a group of patients with locally advanced and/or recurrent tongue base carcinoma. All failed to respond to the conventional treatment modalities or presented so late that were only offered a palliative route. These patients were likely to resist further treatment and are expected to have a very short life expectancy (less than 6 months) with high morbidity.

Here the results showed that the treatment was well-tolerated by all patients and very effective in shrinking tumour and controlling further progression. The great utility of this intervention is not only in its repeatability (unlike radiotherapy) but in the accuracy of treatment. The overall morbidity and mortality after PDT, in this group of patients, were far less when compared with other conventional modalities.

To sum up, the growing body of evidence regarding the successful clinical application of PDT in the management of tissue pathology should encourage clinicians to offer this modality as one of the options when dealing with complicated pathologies. The ability to guide the treatment intra-operatively under ultrasound guidance allows it a leading role in interventional surgical oncology. PDT is a successful modality in the treatment of advanced and/or recurrent tongue base carcinoma. The clinical and radiological end-point parameters are, in fact, very promising for such end-stage disease group.

## Competing interests

The authors declare that they have no competing interests.

## Authors' contributions

All authors have contributed intellectually and to the writing of this manuscript. All authors read and approved the final manuscript.
